# The Influence of a Specialized Dementia Ward on the Treatment of Alzheimer’s Disease Patients

**DOI:** 10.3390/jpm15030082

**Published:** 2025-02-25

**Authors:** Youngsoon Yang, Kyoon Huh, Yong Tae Kwak

**Affiliations:** 1 Department of Neurology, College of Medicine, Soonchunhyang University, Cheonan Hospital, Cheonan 31151, Republic of Korea; astro76@schmc.ac.kr; 2 Department of Neurology, Hyoja Geriatric Hospital, Yongin 17089, Republic of Korea; khuh@ajou.ac.kr

**Keywords:** Alzheimer’s disease, dementia, specialized dementia ward, neuropsychiatric symptoms, discharge rate

## Abstract

**Background:** Hospitalization for severe neuropsychiatric symptoms in Alzheimer’s disease (AD) presents challenges, often requiring environments that ensure safety while addressing therapeutic needs. Traditional closed wards, originally designed for psychiatric conditions like schizophrenia, may not fully address the unique needs of AD patients. This study evaluates the effectiveness of a Specialized Dementia Ward (SDW) tailored for AD patients compared to a General Ward (GW). **Methods:** A retrospective study compared 51 AD patients in an SDW (February 2018–January 2019) and 40 AD patients in a GW (December 2017–January 2018). Patients met NINCDS-ADRDA criteria, with a Clinical Dementia Rating (CDR) ≤ 2 and a Korean Mini-Mental State Examination (K-MMSE) ≤ 20. Clinical assessments at admission and four weeks included K-MMSE, Resident Assessment Instrument Minimum Data Set Version 2.0 (RAI-MDS), and Neuropsychiatric Inventory Questionnaire (NPI-Q). Psychotropic medication use, length of stay, and discharge destination were also analyzed. **Results:** No statistically significant differences emerged between SDW and GW groups regarding baseline demographics, cognitive function, ADL, or neuropsychiatric symptoms. At four weeks, both groups exhibited trends toward improved K-MMSE, RAI-MDS, and NPI-Q scores and reduced psychotropic usage, but these did not reach statistical significance. Although mean length of stay was shorter for SDW patients (3.2 vs. 4.9 months; *p* = 0.078), the difference was not significant. Notably, a significantly higher proportion of SDW patients were discharged home (58.8% vs. 37.5%; *p* = 0.049). **Conclusions:** Although clinical outcomes were comparable, the SDW demonstrated advantages in facilitating discharge to home, suggesting that tailored ward environments may better support AD patients. These findings underscore the importance of therapeutic environments in dementia care and highlight the need for further research on specialized dementia ward designs to improve outcomes and patient satisfaction.

## 1. Introduction

In recent years, the number of Alzheimer’s disease (AD) patients has rapidly increased in South Korea. These patients typically have a chronic disease course, and caregiving and treatment pose significant economic and social challenges. Moreover, many AD patients develop neuropsychiatric symptoms that become too overwhelming for families to manage alone, necessitating hospitalization. Such patients often display severe cognitive impairment and aggressive behaviors, posing threats not only to themselves but also to other patients and caregivers [1].

When hospitalized for neuropsychiatric symptoms, AD patients are often treated with psychotropic medications. However, these treatments tend to have limited efficacy, carry significant side effects, and may even exacerbate neuropsychiatric symptoms [2]. Therefore, reliance on medications alone has clear limitations, highlighting the importance of integrating various non-pharmacological therapies. Another consideration is the need to protect other patients and caregivers from potential harm. Consequently, substantial resources are often required in the long-term, raising important medical and social questions about managing these patients efficiently. One potential solution is the establishment of a Specialized Dementia Ward (SDW) that is similar to closed wards in psychiatric hospitals.

Several studies have explored the benefits of specialized dementia care units or wards, often emphasizing structured environmental modifications, targeted non-pharmacological interventions, and staff training tailored to patients’ cognitive and behavioral needs [3,4,5]. These specialized approaches can reduce neuropsychiatric symptom severity and the use of antipsychotic medications while also improving caregiver satisfaction and overall quality of care. However, the reported effectiveness varies across settings, reflecting differences in ward design, staffing levels, patient mix, and the specific interventions employed [6]. Despite these inconsistencies, a growing body of literature suggests that environments specifically adapted for dementia care can promote safer patient mobility, reduce agitation, and enhance opportunities for therapeutic engagement [3,4,5]. Building upon this evidence, the SDW described in this study was established to address the unique challenges posed by AD patients with severe behavioral symptoms. By creating an environment adapted to the patient’s condition and using environmental (milieu) therapy, this ward offers a tailored setting for dementia care.

In Europe, since the 19th century, institutions were established on the outskirts of towns to isolate individuals with psychiatric illnesses. After World War I, some of these facilities were reorganized into hospitals for mentally ill patients [7]. However, following World War II, most hospitals adopted an “open door” policy, leading to quicker admissions, more active pharmacological treatments, and earlier discharges [8,9,10,11]. In South Korea, dementia patients with psychiatric issues were historically admitted to closed wards in psychiatric departments. More recently, many are admitted to geriatric hospitals that operate wards functionally similar to closed wards, although these are not standardized or unified in concept. Hyoja Hospital, a geriatric hospital admitting many dementia patients, operates a separate SDW for those with severe neuropsychiatric symptoms. Structurally, this ward resembles a traditional closed psychiatric ward, ensuring that patients cannot easily leave the safe areas, but also implements specialized dementia programs. While variations of dementia-dedicated wards exist internationally, little is known about the characteristics of patients admitted to such wards, how the wards are managed, and how they influence patient outcomes. For patients, a key advantage of traditional closed wards is protection from unwanted visitors, thus enhancing their sense of security [12,13,14]. For clinical staff, not having to constantly worry about elopement frees them to focus on the patient’s therapeutic needs, reducing the need for continuous monitoring or physical restraint. Families may also gain peace of mind, knowing that their loved one is in a safer, more stable environment [15]. However, from the patient’s perspective, feeling confined rather than treated can lead to a sense of institutionalization. This power imbalance between caregivers and patients can lead to depression and anxiety in patients. From the caregiver’s perspective, managing access to the ward can be cumbersome or inconvenient, and patients’ resultant emotional distress can negatively affect the therapeutic milieu [16].

Traditional closed wards were not originally developed with dementia patients in mind; they were designed primarily for schizophrenia or substance use disorders and were later applied to various psychiatric conditions. Hence, it is unclear whether this format is beneficial for dementia patients exhibiting challenging behaviors. Nevertheless, because these environments have been used for patients with diverse behavioral issues, they may hold a partial benefit for AD patients who exhibit neuropsychiatric symptoms. However, patients with AD, most of whom experience severe cognitive impairment, differ from other psychiatric patients in several ways. First, they typically have a lower level of insight into their condition. Upon hospitalization, they are more likely to experience separation anxiety due to environmental changes rather than the sense of institutionalization often noted in closed wards [17]. This suggests that such emotional distress may be less pronounced for Alzheimer’s patients in closed wards. Second, because dementia patients are often elderly with multiple comorbidities, they are at higher risk for medical problems such as falls. In open wards, caregivers may resort more frequently to physical or chemical restraints, potentially escalating behavioral disturbances [18]. Paradoxically, the perceived freedom of open wards can actually contribute to a decline in patient mobility, potentially hastening a bed-bound state. While there is a need to restrict the movement of Alzheimer’s patients to a certain extent, it is questionable whether the traditional closed ward model used in psychiatry is necessary. In other words, while traditional closed wards completely restrict the free movement of individuals between the inside and outside of the ward, patients with moderate to severe dementia may have their movement effectively restricted from the inside to the outside with simple devices (such as number keys) due to their cognitive impairment. Thus, while patients cannot freely exit, visitors and caregivers can enter easily, permitting limited active external contact but unrestricted passive external contact.

Hyoja Hospital, a specialized geriatric facility since its establishment, has treated a wide range of elderly patients, including those with strokes, dementia, movement disorders, and terminal cancer [19]. Over 60% have dementia, and most display behavioral problems. Initially, all patients were treated together in open wards. While this approach had several advantages, it also presented challenges, including accidents involving the patients themselves, and conflicts with other patients. This environment also made it difficult to provide intensive observation and treatment. To address this issue, the hospital established a SDW in February 2009 for patients with Alzheimer’s and other dementias who are independently ambulatory but have severe neuropsychiatric symptoms. The SDW combines the strengths of open and closed wards. Unlike a general ward (GW), the SDW was designed with three principles: (1) Restrict patients from leaving the ward, but allow free movement within, minimizing the need for physical restraints commonly used elsewhere. (2) Incorporate environmental therapy by tailoring the ward to patients’ symptoms, enabling them to personalize their rooms with familiar objects and photos. This aligns the living space with the therapeutic space. (3) Provide a direct observation room for patients with severe behavioral disturbances, allowing intensive monitoring and intervention by therapists, thereby minimizing medication and physical restraints.

The primary goal is to manage symptoms so that patients can return home as soon as possible. If this is not feasible, the secondary goal is to transfer them to a nursing home or other care facilities. To assess whether these goals were met, we compared the outcomes of AD patients admitted to the SDW with those in the GW, which is located in the same building with the same structure but operates as an open ward. By comparing changes in cognitive function, activities of daily living, behavioral disturbances, medication usage, length of stay, and the rate of returning home between the two groups, we aimed to investigate whether the ward type had any impact on treatment outcomes.

## 2. Materials and Methods

### 2.1. Participants

The Dementia Clinic at Hyoja Hospital has maintained the Hyoja Hospital Dementia Registry since March 2015, systematically recording clinical and diagnostic data for all dementia patients. This registry includes detailed diagnostic evaluations conducted during patient visits, encompassing medical history, physical and neurological examinations, comprehensive neuropsychological assessments, and routine laboratory tests including ApoE genotyping. Utilizing this registry, the study retrospectively compared AD patients admitted to different wards at different time periods. Specifically, the study screened 54 AD patients admitted to the SDW between February 2018 and January 2019 and 41 AD patients admitted to the GW between December 2017 and January 2018, prior to the establishment of the SDW. After excluding 1 patient from the SDW group and 3 patients from the GW group due to unrelated medical conditions or caregiver-related issues, the final analysis included 51 patients in the SDW group and 40 patients in the GW group. This design allows for a comparative analysis of patient outcomes across two distinct ward environments, leveraging the structured clinical data from the Hyoja Hospital Dementia Registry.

These patients were capable of independent ambulation, displayed primarily behavioral disturbances, and met the NINCDS-ADRDA criteria [20] for AD, with a Clinical Dementia Rating (CDR) [21] score ≤ 2 and a K-MMSE [22] score ≤ 20. Exclusion criteria included the presence of other organic lesions (e.g., brain tumors or trauma) confirmed by at least one MRI or CT scan. The control group consisted of 40 AD patients admitted to GW between December 2017 and January 2018, prior to the SDW’s establishment. Cognitive function was assessed using the CDR-SB and K-MMSE. Activities of daily living (ADL) were assessed with the Resident Assessment Instrument Minimum Data Set Version 2.0 (RAI-MDS) [23], and behavioral disturbances were measured using the Neuropsychiatric Inventory Questionnaire (NPI-Q) [24]. The number of psychotropic drugs prescribed at admission was determined according to the Anatomical Therapeutic Chemical (ATC) classification system. Additionally, antipsychotic dosages were converted to chlorpromazine equivalents [25]. At Hyoja Hospital, cognitive and neuropsychiatric symptoms in AD patients are routinely assessed at 1, 3, 6, and 12-month intervals as part of the hospital’s standard protocol for dementia care. In accordance with this protocol, the study utilized the one-month follow-up assessment to evaluate cognitive and neuropsychiatric symptom scales and medication use after admission. The length of hospital stay was tracked for up to one year.

All participants and their legal representatives were fully informed of the study protocol, and written informed consent was obtained. This study was conducted in accordance with the Declaration of Helsinki and approved by the Institutional Review Board of Soonchunhyang University Cheonan Hospital (IRB No. 2023-02-038-002) on 15 February 2023.

### 2.2. Method

#### 2.2.1. Establishment of the Therapeutic Environment in the SDW and Structural Differences Compared to the GW

Both wards were staffed with 19 nurses and 6 nursing assistants, ensuring comparable levels of care. They were located in the same building but on different floors, each occupying 1785 square meters. While the basic structural layout remained the same, the SDW was specifically designed to accommodate the unique needs of AD patients with severe neuropsychiatric symptoms, incorporating additional therapeutic spaces to enhance patient care. In the SDW, dedicated therapy rooms were established for cognitive rehabilitation and behavioral therapy, providing a controlled environment for structured interventions. The ward also included a direct observation room, allowing continuous monitoring of patients exhibiting severe behavioral disturbances, thus reducing the need for pharmacological or physical restraints. Each patient room in the SDW was designed to promote a familiar and calming environment, incorporating personalized spaces where patients could display photographs, familiar objects, or sensory cues to help reduce anxiety and confusion. Additionally, dining areas, community lounges, and wide corridors with safety rails were included to encourage social interaction and mobility while ensuring patient safety.

Both wards primarily utilized 6-bed shared rooms, but in the SDW, individual spaces were further customized for patients using audio or visual stimuli as part of simulated presence therapy [26]. This approach allowed patients to engage with pre-recorded messages, familiar music, or video content, which could help reduce agitation and reinforce a sense of familiarity in their environment. Unlike traditional closed wards, where patients’ movement is completely restricted, the SDW adopted a semi-restricted approach. The doors to patient rooms were generally kept open, allowing free movement within the ward, while exiting the SDW required entering a simple 4-digit code, preventing unintended wandering without creating an oppressive environment. In contrast to the GW, which primarily relied on a standard ward setup, the SDW was designed to provide a therapeutic living space, ensuring that the physical environment itself contributed to the stability and well-being of dementia patients.

#### 2.2.2. Clinical Outcome Measures

Upon admission, demographic data (e.g., patient’s and caregiver’s age/sex, diagnosis, disease duration, and onset age) were recorded. During the first four weeks of hospitalization, independent neuropsychologists and dementia-specialized nurses (blinded to the study) performed the K-MMSE, RAI-MDS, and NPI-Q assessments. The number of antipsychotic prescriptions and chlorpromazine-equivalent dosages were assessed at admission and again at four weeks. The length of stay and discharge destination (home vs. other facility) were compared between the SDW and GW.

### 2.3. Statistical Analysis

First, independent *t*-tests and chi-square tests were used to compare demographic variables, clinical indices, and length of stay between the GW and SDW. A repeated measure ANOVA was then conducted to identify significant differences in K-MMSE, CDR-SB, RAI-MDS, and NPI-Q at the 4-week follow-up. Analyses were performed using SPSS version 24.0 (SPSS Inc., Chicago, IL, USA).

## 3. Results

### 3.1. Characteristics of the Study Subjects

Of the 54 patients admitted to the SDW and the 41 to the GW, one patient from the SDW and three from the GW were excluded due to unrelated medical conditions or caregiver-related issues (one was excluded due to a newly diagnosed malignancy and three patients were excluded because of family conflicts that resulted in early discharge shortly after admission). The analysis found no significant differences between the groups in demographic information (e.g., sex, age, onset age, disease duration, or education level) or baseline cognitive, ADL, and behavioral measures (Table 1). Both groups averaged two or more psychotropic medications at admission, with chlorpromazine-equivalent doses of 32.7 mg in the GW and 38.5 mg in the SDW, showing no statistical difference (Table 2).

### 3.2. Changes in Cognitive Function, Neuropsychiatric Symptoms and Psychotropic Medication Use Following Admission to Study Wards

All participants were reassessed at baseline and after four weeks. Although the SDW group showed a trend toward greater improvement in K-MMSE, RAI-MDS, and NPI-Q scores, none reached statistical significance. Both groups showed a decrease in the number and dosage of psychotropic medications, but again, these differences were not significant (Table 2).

### 3.3. Length of Stay and Post-Discharge Home Return Rates in the Two Wards

The mean length of stay was 3.2 months in the SDW and 4.9 months in the GW, which did not reach significance (*p* = 0.078). However, a significantly higher proportion of SDW patients were discharged to their homes (58.8% vs. 37.5%, *p* = 0.049) (Table 3). Three patients in the GW were excluded from the final analysis due to pneumonia, fracture, or elopement, and one in the SDW was excluded due to pneumonia (Table 4).

## 4. Discussion

At four weeks post-admission, SDW patients showed somewhat greater (but not statistically significant) improvement in NPI-Q and RAI-MDS scores than GW patients. Since medication usage and dosage did not substantially differ between groups, it is plausible that these trends may be influenced by non-pharmacological or environmental factors, particularly the specialized dementia care environment. Previous research suggests that structured environments, including dementia-friendly architectural design, caregiver training, and routine-based care, can positively impact behavioral and psychological symptoms of dementia and overall patient well-being. The enriched sensory, cognitive, and social stimuli in such environments are known to modulate neuroplasticity and reduce agitation and distress in patients with Alzheimer’s disease (AD) [27]. Additionally, studies indicate that supportive environments can promote residual cognitive functions and maintain the activities of daily living (ADL), thereby potentially modifying disease progression [28]. These mechanisms collectively support the plausibility that environmental influences contributed to the observed trends in our findings.

The length of stay was shorter in the SDW (3.2 months) compared to the GW (4.9 months), though the difference was not statistically significant (*p* = 0.078). However, the rate of returning home after treatment was significantly higher in the SDW at 58.8% compared to 37.5% in the GW (*p* = 0.049). In this study, it is important to confirm how each ward environment (SDW vs. GW) influences the degree of clinical improvement in patients after four weeks of hospitalization. However, what may be even more important is, as demonstrated in this study, the trends observed in the shorter length of stay in the SDW (*p* = 0.078) and the significantly higher likelihood of patients returning home compared to the GW. Although it is difficult to generalize these findings due to variations in patient populations and treatment methods in specialized dementia wards across different countries and institutions [29,30], these results appear to fall somewhere between studies suggesting no significant impact [31] and those showing a significant impact [32].

Interestingly, in the SDW group, there was no significant difference in the length of stay between patients who returned home and those who transferred to other facilities. In contrast, GW patients transferred elsewhere had notably longer stays (4.6 months vs. 6.9 months). This difference might indicate that the GW environment serves more as a de facto long-term care setting, or that caregivers may delay the decision to transfer. Meanwhile, the more specialized SDW environment could prompt earlier discussions and preparations for discharge or transfer. This aspect warrants further research to better understand its implications. The lower dropout rate in the SDW may reflect several benefits of a larger, safer environment, including communal areas that encourage better observation and social adaptation. These findings underscore the need to consider ward design and therapeutic structure, as an over-reliance on medication has well-known limits.

In cases where Alzheimer’s patients exhibit neuropsychiatric symptoms that make home care challenging, hospitalization may be required. Identifying and treating the causes of these neuropsychiatric symptoms are important; however, these issues often do not exist independently but interact with the patient’s living environment. These symptoms may be expressed by their defensive mechanisms against the environment, and conversely, the environment can influence the individual’s ability to cope with these agonizing symptoms. Neuropsychiatric symptoms can be exacerbated or alleviated depending on external environmental factors [33,34]. Additionally, dramatic changes in symptoms or differences in coping abilities among Alzheimer’s patients can result from excessive or diminished responses to external stimuli influenced by the environment. Therefore, it is necessary to adjust environmental factors based on the sensory thresholds of individual patients. While the treatment of Alzheimer’s disease should involve tailoring the environment to the patient’s condition and individual level, most hospitals treat these patients in the same setting as general patients. In such an environment, patients with dementia exhibiting neuropsychiatric symptoms may experience worsening symptoms when hospitalized compared to being treated at home. Due to their reduced ability to adapt to external environments, dementia patients may not benefit from an open ward environment, as these can potentially have a negative impact on their neuropsychiatric symptoms. This study focuses on investigating how a modified closed (or modified open) therapeutic ward, designed to provide an environment that positively influences dementia patients and maximizes safe mobility spaces, impacts the patients’ outcomes. Patients with Alzheimer’s disease often present with various neuropsychiatric symptoms, similar to other psychiatric patients. However, it remains unclear whether treatment spaces like psychiatric closed wards are necessary for these patients. In cases where Alzheimer’s patients with neuropsychiatric symptoms are hospitalized without appropriate clinical expertise or specialized treatment environments, numerous challenges can arise. Therefore, this study aimed to propose a treatment environment model for dementia patients, such as those with Alzheimer’s disease, and to compare it with general wards to identify differences.

However, this study has several limitations. First, it was a retrospective study using historical controls rather than a prospective study with randomized controls for the study group. This is because, from an ethical standpoint, it is challenging to randomly assign hospitalized dementia patients to SDW or GW when a ward specifically designed for dementia patients is available. Nevertheless, during the short study period of one year, there were no groundbreaking advancements in medication for Alzheimer’s disease or its associated behavioral disturbances. Moreover, treatments were provided by the same healthcare providers to both groups, which likely minimized selection bias. Second, there are various confounding variables related to the SDW environment, such as the expertise of caregivers and the involvement of family members, which were not controlled for, potentially introducing confounding errors. In particular, the absence of surveys or interviews with caregivers made it difficult to accurately assess one of the most critical aspects of dementia patient care: the psychological support provided by caregivers.

Despite this study’s limitations—retrospective design, historical controls, a small sample, and potential confounding variables (e.g., family dynamics and staff specialization)—the findings highlight the importance of treatment environments. Future directions for research in specialized dementia wards include larger-scale, multi-center, and ideally randomized controlled trials to validate and generalize these findings in diverse populations. In particular, studies could focus on cost-effectiveness analyses to determine whether the infrastructural investments in SDWs can be offset by reduced hospital stays and lower caregiver burden. Investigations comparing specific non-pharmacological interventions—such as music therapy, occupational therapy, or reminiscence therapy—would help clarify which therapeutic modalities are most beneficial in these specialized settings. Beyond immediate clinical improvements, longitudinal research examining outcomes after discharge (e.g., readmission rates, cognitive trajectories, and caregiver well-being) would offer deeper insights into the long-term value of SDWs. From a practical standpoint, SDWs may evolve further by integrating digital health technologies, such as sensor-based monitoring or virtual reality-based cognitive training, to tailor interventions and enhance safety. Collaboration among neurologists, psychiatrists, nurses, occupational therapists, and social workers should also be systematically studied to optimize staffing models and bolster patient-centered care. Ultimately, establishing consensus guidelines or accreditation systems for SDWs could facilitate more uniform implementation across various healthcare settings, ensuring that best practices are consistently applied to improve both clinical outcomes and patient quality of life.

## Figures and Tables

**Table 1:** Demographic data between patients of General Ward and Specialized Dementia Ward (mean ± standard deviation).

Variables	GW	SDW	*p*-Value *
Numbers (dropout)	41 (1)	54 (3)	0.579
Previous treatment	29 (74.4%)	34 (70.8%)	NS
Sex Male	9	11	NS
Female	30	37	
Age (year)	75.7 ± 7.5	75.3 ± 8.3	NS
Onset age (year)	71.1 ± 8.3	70.6 ± 11.5	NS
Duration (month)	54.9 ± 18.2	56.4 ± 25.4	NS
Education	6.6 ± 3.1	6.9 ± 4.7	NS
K-MMSE	14.4 ± 7.8	13.5 ± 6.5	NS
CDR	1.56 ± 1.1	1.65 ± 0.8	NS
CDR-SB	7.7 ± 5.2	9.0 ± 4.4	NS
RAI-MDS	20.5 ± 11.5	18.4 ± 10.1	NS
NPI-Q	3.0 ± 2.2	4.7 ± 3.4	NS

* Independent *t*-test, Chi-square test was done, GW: General Ward, SDW: Specialized Dementia Ward, K-MMSE: Korean Mini-Mental Sale Examination, CDR: Clinical dementia rating scale, CDR-SB: Clinical dementia scale Sum of Boxes, RAI-MDS: Resident Assessment Instrument Minimum Data Set Version 2.0, NPI-Q: Neuropsychiatric Inventory Questionnaire.

**Table 2:** Comparison of clinical outcomes between General Ward and Specialized Dementia Ward after 4 weeks admission.

	GW (40)			SDW (51)			*p*-Value *
	Baseline	4 Weeks	Delta	Baseline	4 Weeks	Delta	
Psychotropics No	2.5 ± 2.1	2.2 ± 1.2	−0.3	2.1 ± 1.2	2.0 ± 1.0	−0.1	0.857
Equivalent dose	32.7 ± 46.1	24.3 ± 23.6	−8.4	38.5 ± 48.6	37.3 ± 34.4	−1.2	0.492
K-MMSE	14.4 ± 7.8	13.9 ± 8.5	−0.5	13.5 ± 6.5	14.3 ± 6.3	0.8	0.370
RAI-MDS	20.5 ± 11.5	22.1 ± 12.8	1.6	18.7 ± 8.8	17.1 ± 6.2	−1.6	0.148
NPIQ	3.9 ± 2.2	2.6 ± 1.4	−1.3	4.7 ± 4.4	2.5 ± 2.9	−2.2	0.169

* Repeated measure of ANOVA, Equivalent dose; chlorpromazine-equivalent dosage, GW: General Ward, SDW: Specialized Dementia Ward, K-MMSE: Korea Mini-Mental State Examination, RAI-MDS: Resident Assessment Instrument Minimum Data Set Version 2.0. NPI-Q: Neuropsychiatric Inventory Questionnaire.

**Table 3:** Comparison between General Ward and Specialized Dementia Ward.

	GW (40)	SDW (51)	*p*-Value *
LOS (month)	4.9 ± 4.4	3.0 ± 3.0	0.078
LOS in patients with home discharge	1.7 + 0.8	1.8 ± 0.7	0.676
LOS of in patients with other discharge	6.9 ± 6.8	4.6 ± 0.7	0.356
Home discharge (%)	15 (37.5)	30 (58.8)	0.049

* Independent *t*-test, Chi-square test was done, GW: General Ward, SDW: Specialized Dementia Ward, LOS: length of stay.

**Table 4:** Summary of patient withdrawal.

	GW (40)	SDW (51)
Total number of withdrawal (%)	3 (7.5)	1 (1.9)
Serious medical illness (%)	1 (2.5)	1 (1.9)
Fracture (%)	1 (2.5)	0
Abscond (%)	1 (2.5)	0

GW: General Ward, SDW: Specialized Dementia Ward.

## Data Availability

The original contributions presented in this study are included in the article. Further inquiries can be directed to the corresponding author.

## References

[1] Pless A., Ware D., Saggu S., Rehman H., Morgan J., Wang Q. (2023). Understanding neuropsychiatric symptoms in Alzheimer’s disease: Challenges and advances in diagnosis and treatment. Front. Neurosci..

[2] Chang E.S., Rosenberg P.B., Rattinger G.B., Stuart E.A., Lyketsos C.G., Leoutsakos J.M. (2021). Psychotropic Medication and Cognitive, Functional, and Neuropsychiatric Outcomes in Alzheimer’s Disease (AD). J. Am. Geriatr. Soc..

[3] Karrer M., Zeller A., Mayer H. (2023). Dementia care in acute hospitals: A framework for practice development and theory-based evaluation. Nurs. Open.

[4] Chenoweth L., King M.T., Jeon Y.H., Brodaty H., Stein-Parbury J., Norman R., Haas M., Luscombe G. (2009). Caring for Aged Dementia Care Resident Study (CADRES) of person-centered care, dementia-care mapping, and usual care in dementia: A cluster-randomized trial. Lancet Neurol..

[5] Guazzarini A.G., Casanova G., Buchholz F., Kozori M., Lavolpe S., Lichtwarck B., Margioti E., Mendes A., Montandon M.-L., Murasecco I. (2022). The Special Care Unit for People with Behavioral and Psychological Symptoms of Dementia (SCU-B) in the Context of the Project “RECage-Respectful Caring for Agitated Elderly”: A Qualitative Study. Int. J. Environ. Res. Public Health.

[6] Kim S.K., Park M. (2017). Effectiveness of Person-Centered Care on People with Dementia: A Systematic Review and Meta-Analysis. Clin. Interv. Aging.

[7] Murphy E. (1991). After the Asylums: Community Care for People with Mental Illness.

[8] Bowers L., Crowhurst N., Alexander J., Callaghan P., Eales S., Guy S., McCann E., Ryan C. (2002). Safety and security policies on psychiatric acute admission wards: Results from a London-wide survey. J. Psychiatr. Ment. Health Nurs..

[9] Glover G., Kennett C., Quraishi M. (2005). Acute Care 2004: A National Survey of Adult Psychiatric Wards in England.

[10] Rittmannsberger H., Sartorius N., Bradac B., Burtea V., Capram N., Cernak P. (2004). Changing aspects of psychiatric inpatient treatment: A census investigation in five European countries. Eur. Psychiatry.

[11] Zinkler M. (2023). Non-coercive techniques for the management of crises in mental health settings in Germany—A narrative review. Int. Rev. Psychiatry.

[12] Engelhardt J.B., Eisenstein N. (1996). Determination of closed ward placement for chronic geropsychiatric patients: Cognitive and behavioral factors. J. Nerv. Ment. Dis..

[13] Ryan J.H. (1962). The therapeutic value of a closed ward. J. Nerv. Ment. Dis..

[14] Searby A., James R., Snipe J., Maude P. (2023). Locked External Doors on Inpatient Mental Health Units: A Scoping Review. Int. J. Ment. Health Nurs..

[15] Ashmore R. (2008). Nurses’ accounts of locked ward doors: Ghost of the asylum or acute care in the 21st century?. J. Psychiatr. Ment. Health Nurs..

[16] Haglund K., von Knorring L., von Essen L. (2006). Psychiatric wards with locked doors: Advantages and disadvantages according to nurses and mental health nurse assistants. J. Clin. Nurs..

[17] Browne C.J., Shlosberg E. (2006). Attachment theory, ageing, and dementia: A review of the literature. Aging Ment. Health.

[18] Brechtbauer D., Becker C., Eichner B., Koczy P., Nikolaus T. (2005). Factors relating to the use of physical restraint in psychogeriatric care: A paradigm for elder abuse. Gerontol. Geriatr..

[19] Kwak Y.T., Han I.W., Kim D.S., Seo S.H., Lee C.S., Suk S.H. (2000). Clinical characteristics of geriatric patients admitted to Yongin Hyoja Geriatric Hospital. J. Korean Neurol. Assoc..

[20] McKhann G.M., Knopman D.S., Chertkow H., Hyman B.T., Jack C.R., Kawas C.H., Klunk W.E., Koroshetz W.J., Manly J.J., Mayeux R. (2011). The diagnosis of dementia due to AD: Recommendations from the National Institute on Aging–Alzheimer’s Association workgroups on diagnostic guidelines for AD. Alzheimers Dement..

[21] Hughes C.P., Berg L., Danziger W.L., Coben L.A., Martin R.L. (1982). A new clinical scale for the staging of dementia. Br. J. Psychiatry.

[22] Kang Y., Na D.L., Hahn S. (1997). A validity study on the Korean mini-mental state examination (K-MMSE) in dementia patients. J. Korean Neurol. Assoc..

[23] Hutchinson A.M., Milke D.L., Maisey S., Johnson C., Squires J.E., Teare G., Estabrooks C.A., Graham F.A., Kelly L., Burmeister E.A. (2010). The resident assessment instrument—Minimum data set 2.0 quality indicators: A systematic review. BMC Health Serv. Res..

[24] Kaufer D.I., Cummings J.L., Ketchel P., Smith V., MacMillan A., Shelley T., Lopez O.L., DeKosky S.T. (2000). Validation of the NPI-Q, a brief clinical form of the neuropsychiatric inventory. J. Neuropsychiatry Clin. Neurosci..

[25] Woods S.W. (2003). Chlorpromazine equivalent doses for the newer atypical antipsychotics. J. Clin. Psychiatry.

[26] Abraha I., Rimland J.M., Trotta F.M., Dell’Aquila G., Cruz-Jentoft A., Petrovic M., Gudmundsson A., Soiza R., O’Mahony D., Guaita A. (2017). Systematic review of systematic reviews of non-pharmacological interventions to treat behavioural disturbances in older patients with dementia. The SENATOR-OnTop series. BMJ Open.

[27] Day K., Carreon D., Stump C. (2000). The therapeutic design of environments for people with dementia: A review of the empirical research. Gerontologist.

[28] Gitlin L.N., Winter L., Dennis M.P., Hodgson N., Hauck W.W. (2006). A biobehavioral home-based intervention and the well-being of patients with dementia and their caregivers. JAMA.

[29] Mintzer J.E., Golenda C., Wahid L.R., Lewis L., Meeks A., Stuckey M. (1997). Effectiveness of a continuum of care using brief and partial hospitalization for agitated dementia patients. Psychiatr. Serv..

[30] Gonski P.N., Moon I. (2011). Outcomes of a behavioral unit in an acute aged care service. Arch. Gerontol. Geriatr..

[31] Lai C.K., Yeung J.H., Mok V., Chi I. (2009). Special care units for dementia individuals with behavioural problems. Cochrane Database Syst. Rev..

[32] Zieschang T.I., Dutzi I., Müller E., Hestermann U., Grünendahl K., Braun A.K., Hüger D., Kopf D., Specht-Leible N., Oster P. (2010). Improving care for patients with dementia hospitalized for acute somatic illness in a specialized care unit: A feasibility study. Int. Psychogeriatr..

[33] Lawton M.P., Nahemow L., Eisdorfer C., Lawton M.P. (1973). Ecology and the aging process. Psychology of Adult Development and Aging.

[34] Lawton M.P., Simon B. (1968). The ecology of social relationships in housing for the elderly. Gerontologist.

